# Observations on the Nature and Treatment of the More Important Diseases of the Nervous System

**Published:** 1846-01

**Authors:** 


Art. I.—*Observations on the Nature and Treatment of the more important Diseases of the Nervous System.* By a Physician.—*Bath,* 1845. 12mo, pp. 90.

This pamphlet, termed by its author a "Volume," contains a number of remarkable cases of diseases of the nervous system. A short preface is signed "Edward Blackmore, M.D." and dated "Bath, January, 1845." We are at a loss to know why the name of the author was not placed in the title-page, as is usual with authors who court publicity.

There is nothing in this little pamphlet which, in our opinion, merits lengthened criticism. The pathology of the diseases treated by its author as stated by him, appears to us extremely hypothetical. Of apoplexy, he observes, "the immediate cause of a large majority of the cases is a rush of blood to the brain, occasioned by the vehement action of its arteries, and it is probable that this spasmodic action of the vessels continues until, from the failure of the respiratory functions, black blood is circulating in the head; and indeed, even the condition of venous blood being in the arteries of the brain, does not instantly put a stop to the excessive vascular action." (p. 7.) He further observes, "that it is no disproof of this view of the proximate cause of the fit, that sometimes from the moment of its invasion, the pulsation at the temples is low and the face cold; for the capillary branches of the internal carotids are unquestionably sometimes in high action when the branches of the external carotids are in an opposite condition. This view is easily understood upon hydraulic principles, and is confirmed by the fact that, in lessening the determination of blood to the brain, the circulation in the arteries of the head is seen, in these cases, to become excited!" Venous congestion is not, according to our author's views, "the immediate cause of the apoplectic state. . . . The suspension of the functions of the brain appears to be referrible to the *pressure* of the accumulated blood; and partly, also, to *the influence of undecarbonized blood.*" (p. 8.) We have always understood venous blood to be undecarbonized blood; but it appears from the preceding extract, that Dr. Blackmore thinks differently, unless he contradicts himself,—the more probable conclusion.

We observe nothing remarkable in his treatment, except that it appears to be of the most heroic kind, and therefore to be eschewed. Two or three cases of spasmodic affection were treated by a deep crucial incision through the scalp, allowing it to bleed freely. There is nothing, however, in the histories of these cases which would induce the judicious practitioner to adopt the plan as recommended by Dr. Blackmore. He has tried it "several times with gratifying success." "In spasmodic cases of an encephalic origin" he observes, "as well as in hemiplegia and mania, where there has been either a fixed pain in the head or a tender portion of the scalp, so that gentle percussion has induced excessive pain, or a convulsion, or an hysterical fit, my experience, as far as it has gone, has proved that the bleeding from the free incision is a far more powerful remedy than any other mode of depletion." (p. 52.) Two cases of aggravated hysteria were thus treated!

Our note of admiration reminds us of Dr. Blackmore's notes of admiration. They abound, and in conjunction with numerous words in italics mark a succession of medical miracles. "The 26th, *pain in the region of the spine,* the only complaint. *He was then cupped,* and quickly cured. (p. 47.) "The 19th, after the use of the henbane, the left leg became relaxed, *and she could walk, which she had not done for six weeks!*" (p. 59.) "The henbane and purgatives were continued to the 23d, when she was convalescent!" (Ibid.) "The 12th, great and sudden relief after the remedies!" (p. 61.) "In March she was well!" (Ibid.) "— as if an irresistible influence came over her!" (p. 62.) "And sit for some time at the *chaise percée,* without inducing a fit!" (p. 63.) "The uneasy feeling in the head was always relieved on resuming the *horizontal* posture!" (Ibid.) We could fill a page with such quotations. We assure Dr. Blackmore we note these defects with regret; but they are so glaring and exhibit such bad taste as to be altogether inexcusable in a physician.

